# Comparison of different transplantation routes and timing for stem cell therapy in stroke

**DOI:** 10.1080/07853890.2026.2753828

**Published:** 2026-11-03

**Authors:** Qingdong Meng, Hao Deng, Ziqian Zhang, Yongxu Wei, Mingjian Liu, Bin Chen, Yijun Cheng, Hanbing Shang

**Affiliations:** ^a^ Shanghai Institute of Aviation Medicine, Ruijin Hospital, Shanghai Jiao Tong University School of Medicine, Shanghai, China; ^b^ Department of Neurosurgery, Huixian People’s Hospital, Huixian, Xinxiang, China; ^c^ Department of Emergency Medicine, Ruijin Hospital, Shanghai Jiaotong University School of Medicine, Shanghai, China; ^d^ Department of Neurosurgery, Xuanwu Hospital, Capital Medical University, Beijing, China; ^e^ Department of Neurosurgery, Ruijin Hospital, Shanghai Jiaotong University School of Medicine, Shanghai, China; ^f^ Department of Neurosurgery, Huashan Hospital, Fudan University, Shanghai, China; ^g^ Department of Neurosurgery, Ruijin Hospital Luwan Branch, Shanghai Jiao Tong University School of Medicine, Shanghai, China

**Keywords:** Stroke, stem cell, clinical trial, transplantation routes, transplantation timing

## Abstract

**Background:**

Stem cell transplantation holds therapeutic promise for stroke, yet its clinical translation is hindered by unresolved challenges in administration routes, timing, and poor cell engraftment and survival.

**Methods:**

This is a narrative (non-systematic) review. Registered clinical studies were identified in ClinicalTrials.gov, the European Union Clinical Trials Register and the Chinese Clinical Trial Registry, and were supplemented by targeted PubMed searches for the preclinical and mechanistic literature. Records were eligible if they evaluated stem cell transplantation for ischemic or hemorrhagic stroke and reported the delivery route and/or the transplantation time window; records reporting neither were excluded. Because direct head-to-head clinical comparisons of routes and time windows are scarce, registry data and selected preclinical studies were synthesised descriptively rather than pooled, and no meta-analysis was performed.

**Results:**

Each administration route presents a distinct profile. Intravenous injection is minimally invasive and simple but limited by pulmonary sequestration, low cerebral engraftment, and high cell dose requirements. Intra-arterial infusion offers improved targeting but shares similar limitations. Intrathecal delivery allows direct central nervous system access with concentrated doses but carries risks of cerebrospinal fluid leakage and an inflammatory microenvironment. Intracerebral implantation ensures high local engraftment and long-term survival with minimal cell numbers but is invasive and poses risks of hemorrhage and further tissue injury. Intranasal administration is non-invasive with a wide therapeutic window yet exhibits extremely low delivery efficiency. Regarding timing, intravenous and intra-arterial routes are primary in the acute phase; in the subacute phase these routes remain common, alongside intrathecal administration; for chronic stroke, intracerebral and intrathecal transplantation are the predominant strategies in clinical trials.

**Conclusion:**

This review synthesizes two decades of progress and persisting challenges in stem cell therapy for stroke, focusing on delivery strategies and timing. Successful clinical translation will require further large-scale, multicenter prospective studies to optimize these critical parameters.

## Introduction

At present, the treatment methods for ischemic stroke are relatively limited. Both intravenous thrombolysis and arterial thrombectomy have narrow time window limitations and benefit only a small number of patients [1]. Surgical treatment methods, such as craniotomy for haematoma removal, have several disadvantages, including high levels of trauma, high risk and high cost [2]. Moreover, the existing stroke treatment methods cannot repair or improve the damaged neurons or existing neurological dysfunctions. In recent years, a series of clinical trials have shown that stem cells can promote the regeneration of nerve cells and the recovery of damaged neurological functions. However, there are differences and uncertainties in the stem cell therapy administration methods. Animal studies have shown that stem cells improve neurological deficits after stroke. Some of these studies have been verified in clinical trials, achieving favourable results. However, many problems, especially the need to develop technologies to maximize the effectiveness of stem cell therapy on stroke, remain unsolved. Additionally, the optimal cell type, cell dose, transplantation route and candidate patient type urgently need to be clarified [3]. In addition to the scientific improvements, the cell therapy also needs to be evaluated from a commercial perspective. Cell therapy requires the implementation of good manufacturing practice (GMP)-level production methods at a reasonable cost for the production, preservation and transfer of these cells. Herein, we review the current status of stem cell transplantation routes, transplantation timing and existing clinical trials. Moreover, we discuss the prospects of using cell therapy to treat stroke.

The mechanisms of stem cell therapy for stroke include the replacement and bystander effect (BE). When stem cells reach the damaged brain areas within a therapeutic period, they differentiate into new neurons or vascular cells to replace damaged nerves and blood vessels [4]. In addition, stem cells secrete various nutritional factors, such as cytokines, chemokines and exosomes, which can repair damaged neurons, regenerate new neuronal circuits, inhibit inflammatory reactions, mediate immune regulation and promote the regeneration of endogenous stem cells [5]. To date, numerous clinical trials on stem cell therapy for stroke have been conducted worldwide. Nevertheless, these trials did not report any severe adverse events directly associated with the transplantation approach. Importantly, all these trials have demonstrated encouraging clinical outcomes [6,7]. The stem cell transplantation routes mainly include intravenous, arterial, intrathecal, intracerebral, intranasal and intraperitoneal transplantation [7]. Each transplantation route has its own advantages and limitations, indicating that there is no perfect transplantation route. Nevertheless, there are no standards for stem cell transplantation methods in the clinical treatment of stroke.

Moreover, direct head-to-head clinical comparisons of transplantation routes remain scarce. Most studies are small, single-arm or non-randomized and differ in cell product, dose, outcome measures and follow-up, and very few were designed to compare one delivery route, or one time window, against another within an otherwise identical protocol [3,7]. The relative performance of the available routes therefore cannot be established from randomized evidence at present. This gap is the rationale for the present synthesis: the trial registries document which routes and time windows have actually progressed into clinical testing, whereas the preclinical literature provides the only systematic comparisons of engraftment, biodistribution and survival across routes. Both sources are combined and reported separately, so that clinical and preclinical inferences are not conflated.

The choice of route and timing is not arbitrary, because the post-stroke microenvironment evolves over time. In the acute phase (up to 1 week), blood–brain barrier (BBB) disruption, oedema, oxidative stress and a surge of cytotoxic mediators create a hostile niche for grafted cells, while the transiently increased barrier permeability may facilitate the passage of intravascularly delivered cells [8–33]. In the subacute phase (1 week to 6 months), inflammation resolves, endogenous trophic support, angiogenesis and neurogenesis peak, and the BBB is progressively restored, favouring routes that deliver cells into the cerebrospinal fluid or that can be repeated over time [17,34–38]. In the chronic phase (beyond 6 months), a mature glial scar and a largely restored BBB restrict cell entry from the circulation, so that direct intracerebral or intrathecal deposition becomes necessary [39]. These phase-specific constraints underpin the route-by-phase recommendations summarized in Table 1 and in section ‘Timing of Transplantation’.

**Table 1.** Comparison of the advantages and disadvantages of stem cell transplantation methods.

	Intravenous transplantation	Arterial transplantation	Intrathecal transplantation	Intracranial transplantation	Intranasal transplantation	Intraperitoneal transplantation
Advantages	1. Minimally invasive; 2. Low risk profile; 3. Simplified workflow; 4. Repeatable dosing capability	1. Minimally invasive; 2. Higher stem cell homing to injured brain regions compared to IV route	1. Minimally invasive; 2. Enables high-concentration stem cell delivery to injury site; 3. Uniform distribution	1. Highest engraftment rate; 2. Requires minimal cell dose (10^1–10^ cells); 3. Prolonged survival in injury niche (up to 2 years) [40]	1. Noninvasive, rapid procedure; 2. Extended therapeutic time window	Minimally invasive
Disadvantages	1. Pulmonary first-pass elimination effect; 2. Short intracerebral survival (∼24 h); 3. High cell dose requirement (10^8^–10^9^ cells); 4. Low engraftment efficiency	1. Hepatic first-pass elimination effect; 2. Prone to ischemic brain injury; 3. Short intracerebral survival (∼24 h); 4. High cell dose requirement (10^8^ to 10^9^ cells); 5. Low engraftment efficiency	1. Risk of hydrocephalus/cerebrospinal fluid leakage; 2. Suboptimal microenvironment for stem cell survival	1. Hemorrhagic risk; 2. Catheter occlusion risk; 3. Cell suspension jetting risks; 4. Hostile microenvironment for survival	1. Gastrointestinal first-pass elimination effect; 2. Ultralow delivery efficiency (0.3% cell retention)	Low engraftment efficiency

IV: intravenous; IA: intra-arterial; IT: intrathecal; IC: intracerebral; IN: intranasal; IP: intraperitoneal; BBB: blood–brain barrier.

## Materials and methods

This article is a narrative (non-systematic) review and was prepared and reported with reference to the Scale for the Assessment of Narrative Review Articles (SANRA). Four sources were searched: PubMed, for peer-reviewed clinical and preclinical reports; and ClinicalTrials.gov, the European Union Clinical Trials Register (clinicaltrialsregister.eu) and the Chinese Clinical Trial Registry (chictr.org.cn), for registered clinical studies irrespective of publication status. In the registries, the condition or disease field was searched with the terms ‘cerebral infarction’, ‘stroke’, ‘cerebral hemorrhage’ and ‘intracerebral hemorrhage’, and the intervention or title field with ‘stem cell’; each condition term was combined with the intervention term in a separate query. In PubMed, terms were combined as (‘ischemic stroke’ OR ‘hemorrhagic stroke’ OR ‘cerebral infarction’ OR ‘intracerebral hemorrhage’) AND (‘stem cell’ OR ‘mesenchymal stem cell’ OR ‘neural stem cell’ OR ‘bone marrow mononuclear cell’ OR ‘progenitor cell’) AND (‘transplantation route’ OR ‘intravenous’ OR ‘intra-arterial’ OR ‘intrathecal’ OR ‘intracerebral’ OR ‘intranasal’ OR ‘intraperitoneal’ OR ‘timing’ OR ‘time window’). Records were screened by title and registry summary, and the full record or full text was examined whenever eligibility was unclear. The final search was completed on 12 December 2025, and no language restriction was applied.

Records were included if they (1) enrolled patients with, or used an animal model of, ischemic or haemorrhagic stroke; (2) administered stem or progenitor cells of any tissue origin; and (3) reported either the delivery route or the interval between stroke onset and cell administration, or both. Records were excluded if they (1) administered only cell-derived products, such as exosomes or conditioned medium, rather than cells; (2) reported neither the route nor the timing, so that no route- or phase-specific inference could be drawn; or (3) were duplicate registrations of the same trial, in which case the most recently updated record was retained.

From each clinical record, the registration number, stroke type, administration time window, cell type, dose, delivery route and recruitment status were extracted. From each preclinical study, the animal model, method of stroke induction, administration timing, cell type, dose and delivery route were extracted. Because the included records differed in design, cell product, dose, route, timing and outcome measures, and because direct comparisons were scarce, the data were synthesized descriptively. No effect estimates were pooled and no meta-analysis was undertaken.

### Transplantation routes

#### Intravenous transplantation

Intravenous administration of stem cells means injecting therapeutic cells systemically through the bloodstream. These stem cells then travel through the venous system to migrate the cerebral injury sites for subsequently targeted neuronal repairment and functional recovery [41–43]. By inputting ‘cerebral infarction’, ‘stroke’ and ‘intracerebral hemorrhage’ in the condition/disease search box on clinicaltrials.gov (a US clinical trial registration website) and inputting ‘stem cell’ in the intervention/treatment search box for each search, the results revealed that a total of 16 clinical trials on the venous transplantation of stem cells for stroke treatment were completed and that seven clinical trials are in the recruiting stage. In addition, three clinical trials are not yet recruiting. Among all the retrieved clinical trials, only one trial focused on the treatment of haemorrhagic stroke, and the remaining trials focused on the treatment of ischemic stroke. By inputting the words ‘cerebral infarction stem cell’, ‘stroke stem cell’, ‘intracerebral hemorrhage’ and ‘stem cell’ in the search box on clinicaltrialsregister.eu/ctr-search/search (a European Union clinical trial registration website), the results revealed only one ongoing clinical trial on the venous transplantation of stem cells for the treatment of ischemic stroke. By inputting the words ‘cerebral infarction’, ‘cerebral hemorrhage’ and ‘stroke’ in the research disease search box on chictr.org.cn (a Chinese Clinical Trial Registration Center website) and inputting ‘stem cell’ in the registration title search box for each search, the results revealed three clinical trials on the venous transplantation of stem cells for the treatment of ischemic stroke. Notably, one trial is not yet recruiting for haemorrhagic stroke, and two trials have been completed for ischemic stroke.

Among the clinical trials on the stem cell transplantation for stroke treatment, the number of clinical trials via the venous route was predominant. The administration of stem cells to the pathological regions via the circulatory system is a quantitatively validated therapeutic modality [44–46]. It has the following advantages: ① it serves as a customary clinical operation, resulting in the slightest adverse effects on the body; ② it demonstrates superior technical accessibility demanding only venipuncture devices, which significantly decreases the incidence of procedural complications [47,48]; ③ stem cells are transported via the circulatory system throughout the body, enabling them to reach haemorrhagic lesions in the brain parenchyma as well as secondary injuries in remote organs, such as the spleen, intestines and lungs. Consequently, it ameliorates the multi-organ homeostasis secondary to a stroke; and ④, intravenous administration is minimally invasive, facilitating serial dosing [49].

However, regarding the advantage of the jugular vein transplantation route, contradictory conclusions exist. Mello et al. [46] reported that mesenchymal stem cells (MSCs) administered intravenously have no obvious effect on the treatment of haemorrhagic stroke. Chen et al. [45] indicated that delivering MSCs intravenously decreased the level of ONOO(−), while ameliorating the physiological integrity of BBB in rats with haemorrhagic stroke, thereby, accelerating neurological rehabilitation. This discrepancy could arise from the methodological differences in Chen et al.’s study, including the use of elevated stem cells dosage, earlier intervention timing after stroke, and a reduced infusion rate during administration. However, the specific mechanism responsible for these phenomena remains to exploration.

In addition to the above advantages, venous transplantation also has disadvantages. Only a small number of stem cells were found in damaged lesions, while most stem cells were trapped in the lungs [50]. The pulmonary retention rates of stem cells post-intravenous administration were approximately 50–60% [51]. The inhibitory effects of apoptosis and inflammation caused by the BE of neurotrophic factors are considered as the main mechanism of the venous transplantation of stem cells [52]. This phenomenon was obviously observed in the acute phase of ischemic stroke, but not in the chronic phase [50,51]. In terms of the timing of stem cell transplantation and the number of stem cells used, venous transplantation and arterial transplantation are more appropriate transplantation routes in the acute and subacute phases of stroke [53]. Notably, prior studies have shown that the number of cells used for the venous transplantation of stem cells is ∼10^9^ [51].

Extensive clinical trials on intravenous stem cell transplantation for stroke treatment have been conducted globally, with its advantages being widely acknowledged by multiple domestic and international research institutions. In the future, more corresponding clinical trials need to be conducted to solve the inherent first-pass elimination problem of venous transplantation or to develop corresponding auxiliary transplantation methods to increase the implantation rate of venous transplanted stem cells in the injured brain areas.

#### Arterial transplantation

Arterial transplantation of stem cells refers to the method of delivering stem cells to the injured brain area by arterial injection or the use of a catheter to guide stem cells into the carotid artery or the circle of Willis in the brain [54,55]. According to the above search methods in terms of arterial transplantation of stem cells for treating stroke, the data retrieved from the clinicaltrials.gov website revealed four completed clinical trials and one clinical trial in the not recruiting stage, with each trial only focusing on ischemic stroke. Notably, no relevant clinical trials of arterial transplantation of stem cells for treating stroke were retrieved from the clinicaltrialsregister.eu/ctr-search/search website. On the chictr.org.cn website, only one clinical trial of arterial transplantation of stem cells for treating ischemic stroke was retrieved, and this clinical trial is not yet recruiting.

In cerebral ischemia and traumatic injury, administration stem cells via intra-artery demonstrated enhanced homing efficiency to the lesioned brain regions when compared with the intravenous administration [55–57]. Guzman et al. demonstrated that fluorescence-activated sorting of neural stem cells (NSCs) for CD49d followed by intracarotid delivery enhances NSC homing to ischemic lesions and improves functional recovery in mice [58]. Extending this approach to the cellular level, Jablonska et al. showed that forced expression of very late antigen-4 (VLA-4) in glial-restricted progenitors increased adhesion to VCAM-1-coated surfaces and TNFα-activated endothelium, while also enhancing chemotactic migration. Importantly, intra-arterially infused VLA-4^+^ GRPs exhibited a threefold increase in vascular adhesion and improved diapedesis, as confirmed by multiphoton imaging [59].

Real-time imaging has further refined the precision of intra-arterial stem cell delivery. Using MRI, Walczak et al. visualized the successful arterial delivery of superparamagnetic iron oxide (SPIO)-labelled MSCs – an achievement not possible with intravenous routes [60]. In acute stroke, however, intra-arterial infusion yielded variable engraftment, which correlated strongly with intra-infusion laser Doppler flow (LDF) reductions (up to 80%). Notably, severe LDF drops predicted poor cell homing and heightened morbidity, highlighting the need for real-time monitoring. Complementing this, high-speed GE-EPI MRI enabled real-time tracking of SPIO-labelled cells during intra-arterial delivery across species, from rats to naive dogs and swine [61]. Beyond localization, SPIO contrast confirmed vascular patency post-infusion. Together, these advances establish real-time MRI as a powerful tool to optimize intra-arterial targeting, improve safety and support clinical translation, particularly relevant for human-scale brains.

However, several safety concerns are worthy of attention. For example, embolic events, haemorrhage and infection, etc. restrict the wide-scale application of this administration approach [62]. Some studies have shown that arterial delivery of stem cells was not effective for the brain transplantation of stem cells. In general, arterial transplantation for stroke therapy has the following limitations: ① the cells that clog the artery can cause additional ischemic injury [63]; and ② most of the stem cells transplanted through arterial routes are not in the brain but exist in other remote organs, such as the lungs, spleen and bladder [54]. Compared with the intravenous stem cell transplantation, arterial stem cell transplantation results in significantly higher radioactivity accumulation rates in the liver (2 h: 40% vs. 14%; 24 h: 47% vs. 19%) and spleen (2 h: 6% vs. 2%; 24 h: 7% vs. 3%), but lower accumulation rates in the lungs (2 h: 7% vs. 21%; 24 h: 4% vs. 7%) [64]. Thus, the distributions of stem cells transplanted through veins and arteries were different shortly after transplantation. Most stem cells transplanted intravenously appear in the lungs, while stem cells transplanted intra-arterially appear in the liver [50]. Another study has reported that even almost no bone marrow-derived mononuclear cells (BMMNCs) are detected in the brain after 24 h, but in other organs [65].

Despite concerns regarding embolic complications associated with intra-arterial stem cell delivery, emerging evidence indicates that such risks are not intrinsic to the route itself but are highly dependent on procedural parameters. In a rat middle cerebral artery occlusion (MCAO) model, Yavagal et al. demonstrated that low-dose intra-arterial infusion of MSCs (1 × 10^5^ cells) preserved cerebral blood flow and exerted neuroprotective effects when administered in the subacute phase [66]. Conversely, Cui et al. reported that escalating allogeneic bone marrow-derived mesenchymal stem cell (BM-MSC) doses (0.25–1.0 × 10^6^ cells) progressively compromised perfusion, enlarged infarct volumes and aggravated sensorimotor deficits. Notably, even a slow infusion rate (0.5 mL over 6 min) failed to prevent complications, with histopathological analyses confirming necrotic tissue loss and BBB disruption [67]. These findings underscore that optimization of cell dose and infusion kinetics, rather than categorical avoidance of intra-arterial delivery, is critical for safe clinical translation.

These results indicate that stem cells transplanted through veins or arteries do not stay in the brain for a long period of time. Although the intra-arterial injection avoids the first-pass effect caused by pulmonary microvessels, it is currently uncertain whether stem cells with a relatively large diameter effectively penetrate the BBB and effectively reach the lesion [68]. Currently, relatively few clinical trials on the arterial transplantation of stem cells for the treatment of stroke exist. Like the venous transplantation, arterial transplantation also has a first-pass elimination effect. In the future, more and larger-scale clinical trials are needed to solve this problem and prolong the survival time of transplanted stem cells in the injured brain areas.

#### Intrathecal transplantation

Intrathecal transplantation refers to the method of delivering stem cells into the subarachnoid space through lumbar puncture or ventricular puncture. Cerebrospinal fluid circulation transports stem cells to the damaged brain areas [69–71]. Intrathecal injections of autologous or allogeneic MSCs have been used to treat various human neurological diseases, such as cerebral palsy [72], progressive multiple sclerosis [73], spinal cord injury [74], amyotrophic lateral sclerosis [75], amyotrophic lateral sclerosis [76], progressive supranuclear palsy [77], cerebral haemorrhage [78], spinocerebellar ataxia [79] and multiple system atrophy [80]. All these above studies revealed no serious stem cell-related adverse complications. Moreover, most patients included in these studies had improved outcomes.

According to the above search methods, the data retrieved from the clinicaltrials.gov website revealed only two clinical trials on the intrathecal transplantation of stem cells for treating ischemic stroke. These clinical trials are marked as completed. On the clinicaltrialsregister.eu/ctr-search/search website, no relevant clinical trials on the intrathecal transplantation of stem cells for treating stroke were retrieved. The data on the chictr.org.cn website revealed two clinical trials on the intrathecal transplantation of stem cells for treating ischemic stroke, including one that is recruiting and one that is completed.

Intrathecal injection is a safe and feasible method [81–83]. Injecting stem cells into the patient’s subarachnoid space through lumbar puncture provides an effective way for stem cells to cross the BBB. This approach circumvents both the first-pass metabolism and splenic phagocytosis [84]. Thus, the grafted cells and their secreted nutritive factors can mediate direct healing effects on the lesion site. Intrathecal transplantation has the following advantages: ① intrathecal injection allows a high concentration of stem cells to directly migrate to the lesion site [69]; and ② compared with the intracerebral injection, intrathecal injection may lead to better stem cell distribution, which is safer and less invasive [85]. However, there are still several unresolved problems associated with the intrathecal administration as follows: ① the concrete implantation rate of stem cells administered intrathecally is unknown, which may result in hydrocephalus and cerebrospinal fluid leakage [86]; and ② the hostile lesional microenvironment characterized by the inflammation, acidosis and hypoxia severely may compromise the viability of engrafted stem cells [84,87]. The multimodal integration of haematoma evacuation, hyperbaric oxygenation and genetic engineering synergistically augments the viability of engrafted stem cells and amplifying the treatment benefit [51]. The unique advantages of intrathecal transplantation have been verified in many clinical trials. In the future, more and larger-scale clinical trials are still needed to explore the implantation rate of stem cells via the intrathecal transplantation route, methods to avoid related complications, and methods to improve the survival rate of transplanted stem cells in the damaged brain areas.

#### Intracerebral parenchymal transplantation

Intracerebral parenchymal transplantation refers to the direct delivery of stem cells to damaged brain areas by puncturing the brain parenchyma [88–90]. According to the above search methods and the search keywords used for separate searches, the data retrieved from the clinicaltrials.gov website revealed six completed clinical trials related to the treatment of ischemic stroke. One clinical trial related to the treatment of ischemic stroke is in the recruiting stage, another one clinical trial related to the treatment of haemorrhagic stroke is in the unknown stage. The data retrieved from the clinicaltrialsregister.eu revealed one completed trial for the treatment of ischemic stroke. The data retrieved from the chictr.org.cn website revealed three related clinical trials on stem cell transplantation for ischemic stroke, including one that is completed, two that are not yet recruiting, and one that is recruiting.

The advantages of stem cell transplantation in the brain parenchyma are as follows: ① intracerebral administration delivers more stem cells to the damaged brain area and achieves the highest stem cell implantation rate [91]; ② the number of stem cells used for stem cell transplantation in the brain parenchyma is only ∼10^7^ cells, which is less than that used for vascular transplantation of stem cells (∼10^9^ cells) [51,92]; ③ intracerebrally injected iron-labelled BM-MSCs could effectively migrate, settle in ischemic brain areas and survive for more than two years [40], which is longer than the 24 h survival time for the vascular transplantation route [50,51,93]; ④ direct delivery of stem cells into the brain parenchyma can circumvent the BBB, while bypassing pulmonary first-pass metabolism and splenic phagocytic filtration [94–96]. This localized administration enables these transplanted stem cells and their secreted neurotrophic factors to accumulate preferentially at the injury sites [97,98]. Thereby, optimizing their regenerative potential and therapeutic efficacy in the neurological pathologies [99]. However, this stem cell transplantation in the brain parenchyma also has several disadvantages. First, the transplantation cannula may cause additional brain damage, including the cerebral haemorrhage caused by vascular damage, and brain parenchymal cell debris or cell clusters may block the cannula (most stem cells are adhesive), therefore, causing stem cell death or suspension jetting [100]. Prior studies [51] reported that one case of subdural haematoma occurred in 18 patients who underwent brain parenchymal stem cell transplantation using the Pittsburg cannula. Another study reported that one case of subdural haematoma occurred in 13 patients. In this study, one quarter of the cannulas were blocked during the cell survival check and that abundant brain tissue debris was found when the Pittsburg cannula was used. In addition, among the seven patients who underwent stem cell transplantation using the Mizuho cannula, three patients experienced cannula blockage. To encounter the cannula blockage, surgeons may push the syringe slightly harder or change the cannula. When pushing the syringe, it is difficult to break up cell clusters, which may cause a strong force on the cells or the cell suspension, resulting in stem cell death or substantial jetting of the cell suspension. Considering that jetting is essentially used for brain tumour resection, jetting the cell suspension may in turn cause additional damage to the brain parenchyma. Second, similar to the intrathecal transplantation, the hostile lesional microenvironment characterized by inflammation, acidosis and hypoxia severely compromises the viability of engrafted stem cells [85,101]. The integration of haematoma evacuation, hyperbaric oxygen therapy, genetic engineering strategies ameliorates the engrafted stem cells viability and amplifies the therapeutic outcome. Direct administration of stem cells into the cerebral parenchyma requires meticulous procedure to prevent iatrogenic complications, such as the intracranial infection, tumorigenesis and cerebral herniation [85]. In the future, more and larger-scale clinical trials are still needed to solve the problem of damage to brain parenchymal cells.

#### Intranasal transplantation

Intranasal transplantation refers to the introduction of a stem cell suspension deep into the nasal cavity using a polypropylene catheter. The tip of the catheter is placed in the olfactory epithelium area at the back of the nasal cavity. The stem cell suspension is slowly pushed into the catheter using a 10 μL glass syringe connected to the catheter (animal experiment) [102].

There are relatively few clinical trials on the intranasal transplantation of stem cells for stroke treatment. According to the above retrieval methods and keywords, data retrieved from the clinicaltrials.gov website revealed one completed clinical trial, three clinical trials in the recruiting status, and one clinical trial in the not yet recruiting stage, with both trials focusing on the treatment of ischemic stroke. No relevant clinical trials on the intranasal transplantation of stem cells for stroke treatment were retrieved from the clinicaltrialsregister.eu website. Four clinical trials on intranasal transplantation of stem cells for ischemic stroke were retrieved from the chictr.org.cn website, including one that is recruiting, one that is completed, and two that are not yet recruiting. In addition, one clinical trial designed for haemorrhagic stroke is not yet recruiting.

Transnasal stem cell transplantation has become a new treatment strategy with the following advantages: ① it was a noninvasive administration modality, which can execute time-efficiently [98,102,103]. The olfactory epithelium mediated trans-nasal delivery allows stem cells to bypass the nasal mucosa barrier, with subsequent migration into the cerebral parenchyma occurring through the olfactory neural projection or cerebrospinal fluid pathway, thereby ameliorating the therapeutic efficacy of stroke [104]; ② trans-nasal transplantation of MSCs has a wide therapeutic window, which can lead to long-term improvements in the sensorimotor and cognitive functions, as well as reduced damage to the white and grey matter after ischemia [105,106]. MRI and immunohistochemical analyses in the animal experiments have revealed that MSCs could reach the targeted lesion sites within only 2 h after intranasal administration. At 12 h after administration, the number of MSCs at the lesion site reached a peak and significantly decreased at 72 h [51]. Following the MSCs administration, MSCs migrated into the brain and localized around the cerebral vessels in the ipsilateral cortex on day 1 after intranasal delivery, enhanced pial collateral remodelling, accelerated blood flow recovery in the peri-infarct regions, reduced infarct volume and cortical tissue loss, and promoted functional recovery after stroke on days 7, 14 and 21 post-transplantation [107]. However, this intranasal transplantation still has several disadvantages. For example, in terms of efficiency, this method was markedly inferior to the stereotaxic intracerebral administration [108,109]. Post-administration biodistribution analyses revealed substantial diversion of transplanted stem cells to the gastrointestinal tract, with only merely 0.3% successfully traversing the nasal mucosa to ultimately reach cerebral parenchyma [50]. While hyaluronidase co-administration may elevate the transmigration ratio by around twofold. However, the quantitative analyses failed to demonstrate statistically the meaningful enhancement in cellular engraftment within targeted neuroanatomical structures, particularly the hippocampus, thalamus and cortical regions [50,110].

#### Intraperitoneal transplantation

Intraperitoneal transplantation refers to the direct injection of stem cells into the abdominal cavity, using the ability of the peritoneum to absorb stem cells into the damaged brain area [85,111]. No relevant clinical trials on the intraperitoneal transplantation of stem cells for stroke treatment were retrieved from all the clinicaltrials.gov, clinicaltrialsregister.eu and chictr.org.cn websites. Animal experiments have shown that intraperitoneal injection of human MSCs leads to rapid aggregation of cells in the abdominal cavity, and only a small number of cells can migrate to other places [50]. Compared with the intravenous injection of MSCs, intraperitoneal injection resulted in less aggregation of stem cells in the peripheral tissues (such as the lungs and liver) [112]. However, it is difficult to detect stem cells in the ischemic frontal cortex after intraperitoneal transplantation of umbilical cord mesenchymal stromal cells. Marzban et al. investigated the efficacy of intraperitoneally administrated bone marrow MSCs in multiple sclerosis therapy, their finding revealed no detectable bone marrow MSCs migration to the corpus callosum, demonstrating the limited cellular penetration into the cerebral lesion sites via this delivery route [111]. The advantage of this intraperitoneal transplantation is that it results in less trauma. However, the disadvantage is that the transplantation efficiency is low [85]. At present, only few animal experiments have used intraperitoneal transplantation to treat stroke, and no relevant human experiments have been performed.

A comparative summary of the advantages and disadvantages of various stem cell transplantation methods described above is presented in Table 1. Furthermore, a schematic diagram was provided to illustrate the routes of stem cell administration for clarity (Figure 1).

**Figure 1.**
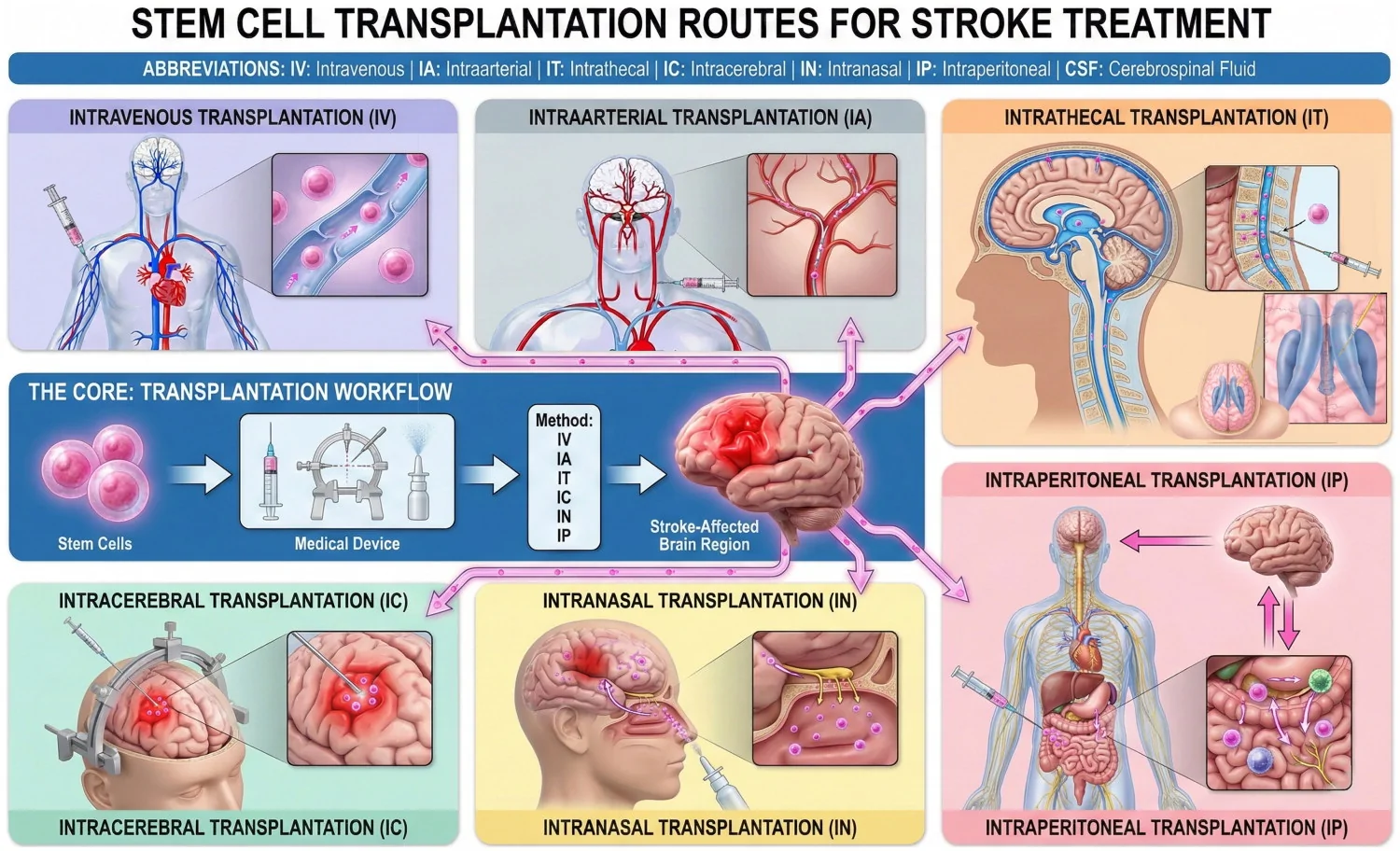
A schematic diagram illustrating the routes of stem cell administration. IV: intravenous; IA: intra-arterial; IT: intrathecal; IC: intracerebral; IN: intranasal; IP: intraperitoneal.

#### Timing of transplantation

In addition to the above transplantation routes, the timing of stem cell transplantation is likewise crucial in the treatment of stroke. The temporal coordination of transplantation procedures significantly determines the viability of stem cells. There is currently no consensus on the optimal timing of stem cell transplantation for achieving the best treatment effect. Temporal alterations in the post-transplantation microenvironment critically modulate the viability of engrafted stem cells. For example, 8–12 h after intracerebral haemorrhage, the permeability of the BBB increases significantly, making it easier for stem cells to penetrate the barrier and enter the brain tissues [113]. However, post-haemorrhagic cerebral tissues exhibit transient elevations of inflammatory mediators (TNFα and IL-1β, etc.) in the peri-haematoma regions, with cytokines concentrations peaking 6–72 h following stroke onset [114,115]. These neurotoxic mediators in the acute phase induce iron overload, metabolic dysfunction, apoptotic activation, collectively and impairing grafted stem cells viability [116]. It induces the stem cells depletion, thereby compromising neuronal restoration and regenerative processes. However, this does not mean that delaying transplantation will lead to greater benefits. If the inordinate postponement of neuron restoration intervention predisposes patients to global cerebral compromise, it may result in irreparable neural circuitry loss [117]. The temporal delay in transplantation consistently coincides with two concurrent processes: BBB restoration and diminished level of neuroprotective factors, potentially compromising stem cells homing efficiency [3,118]. However, a meta-analysis of 40 preclinical studies on haemorrhagic stroke showed that the intracerebral administration demonstrates superior therapeutic outcome in ameliorating functional deficits and parenchymal injury, with delayed intervention exceeding seven days post-ictus achieving maximal neuroprotective efficacy against haemorrhagic stroke-induced secondary damage [119]. Research [50] on the stem cell treatment for haemorrhagic stroke has focused on a range of possible transplantation time points from the haemorrhagic event onset through 60 days post-ictus. Thus, the optimal temporal window for attaining maximum therapeutic efficacy via stem cell transplantation in haemorrhagic stroke remains undetermined. Because most of the current clinical trials investigating stem cell therapy for stroke have focused on ischemic stroke, the following staging of stem cell transplantation will address this specific subtype of stroke.

#### Acute-phase transplantation

Stem cell transplantation within one week after stroke onset is defined as acute-phase treatment [120]. Because it is difficult to expand autologous MSCs *in vitro* during this period, BMMNCs or allogeneic stem cells are selected. These trials used intravenous or arterial transplantation instead of intracerebral injection [51]. Although earlier studies with small samples and no control groups have reported good clinical outcomes, two studies on intravenous and arterial stem cell transplantation with control groups have reported no statistically significant differences in the functional recovery between the stroke and control groups [110,121]. The negative efficacy of this intervention method may be associated with the therapeutic time window (24–48 h) after stroke [121]. Notably, a clinical study is currently being conducted on bone marrow-derived monocyte transplantation for ischemic stroke under a novel time window framework (24–36 h post-enrolment) [122].

#### Subacute phase transplantation

Stem cell transplantation for stroke between 1 week and 6 months is considered as subacute phase treatment. In addition to the BMMNCs, autologous bone marrow MSCs can also be used for *in vitro* expansion at approximately 1 month. Although intravenous or arterial transplantation accounts for most of these trials, intrathecal transplantation has also been reported [50,51]. The results of these studies were similar to the favourable results reported in small sample trials used in the acute phase. However, larger-scale randomized trials have shown different outcomes. In a prior study [123], half of the patients received approximately 3 × 10^7^ autologous BMMNCs within 7–10 days after injury. This phase II multicentre, parallel-group, randomized, blinded trial revealed that although the intravenous infusion of BMMNCs was safe, it had no beneficial effects on the stroke outcomes. Intravenous injection of 1 × 10^8^ bone marrow MSCs achieved better recovery and reduced mortality within 5 years after the start of treatment when compared with the controls [124]. Differences in cell processing procedures, patient types and timing make it more difficult to draw specific conclusions. Moreover, the results of arterial transplantation treatment also differ in various trials. Bhatia reported a good outcome of recovery through arterial transplantation of BMMNCs, whereas others have not reported differences [5].

#### Chronic-phase transplantation

Stem cell transplantation treatment starting 6 months after the occurrence of ischemic stroke is considered as the chronic phase of treatment [51]. Currently, there is no effective treatment method available for this stage. During this period, the intracerebral or intrathecal transplantation routes are chosen by most clinical studies. However, only one clinical study on the intracerebral transplantation of stem cells for stroke treatment used control patients (this trial included six patients with cerebral infarction and four patients with cerebral haemorrhage). One study reported that patients who received transplantation of CD34-positive hematopoietic stem cells via the intracerebral transplantation route had significant neural recovery when compared with the control group [123]. The results of intravenous transplantation vary across different trials. Some studies [5,110] reported significant recovery when compared with the control group, whereas others did not. Randomized clinical trials using larger patient samples are currently ongoing (NCT02448641 and NCT02448641).

Stem cell transplantation has become a promising treatment approach for stroke. An increasing number of empirical studies have shown that stem cell transplantation has undeniable potential in stroke treatment, as it reduces neuroinflammation, accelerates angiogenesis, accelerates neurogenesis and promotes functional recovery. Moreover, the safety of stem cell transplantation has been verified through many studies, thus paving the way for the clinical application of stem cells [74]. Most methods for stem cell transplantation seem safe. In the acute stage, intravenous or intra-arterial transplantation is the preferred option. While, intracerebral transplantation is preferred in the chronic stage. At this time, cell implantation is considered the purpose of cell therapy itself. Optimized treatment of stroke remains an urgent and unmet need. The evolving evidence not only emphasizes the potential of stem cell therapy for stroke, but also highlights the challenges that must be overcome to achieve continuous and effective treatment for patients [7,125]. At present, there is no unified understanding or standard worldwide regarding the selection of stem cell transplantation routes and the timing for stroke. There is currently a lack of comparisons of the therapeutic effects and efficiencies of different stem cell transplantation routes for stroke treatment. Moreover, there is no corresponding clinical trial on the relationship between the types and stages of stroke and stem cell transplantation routes. In the future, both preclinical and clinical studies exploring the efficiency of stem cell transplantation routes and timing of different types and stages of stroke are urgently needed.

## Conclusions

This paper summarized the stem cell transplantation methods and timing for the treatment of stroke, introducing the advantages and disadvantages of each transplantation route and timing. In addition, we narratively reviewed the theoretical and technical progress and shortcomings of stem cell treatment for stroke over the past 20 years. To provide a comprehensive overview, we have narratively summarized clinical studies on both haemorrhagic and ischemic stroke, with extracted details including clinical trial registration numbers, disease type, administration time window, stem cell classification, dosage, delivery route and recruitment status (Table 2). Furthermore, key preclinical studies have also been categorized, detailing experimental parameters such as animal models, disease induction, administration timing, stem cell type, dosage and delivery route (Table 3). Overall, this review is descriptive. It synthesizes heterogeneous registry records and selected preclinical studies, most of which are small, non-randomized and subject to confounding between route, timing, cell type and dose, and it does not generate new primary data or pooled effect estimates. The staged recommendations on route and timing given above should therefore be regarded as hypothesis-generating, and as a framework for designing randomized, multicentre, head-to-head trials, rather than as definitive guidance for clinical practice.

**Table 2.** Registered clinical trials.

Clinical trial registration number	Disease	Administration time	Classification of stem cells	Dose	Route	Recruitment status
*ClinicalTrials.gov (* https://clinicaltrials.gov *)*						
NCT06752720	Chronic ischemic stroke	Between 6 months and 5 years after symptom onset	Autologous mesenchymal stem cells	4 × 10^7^ cells	IC	Recruiting
NCT03570450	Ischemic stroke	Within 7 days of symptom onset	Adipose derived stem cells	1 × 10^6^ to 3 × 10^6^ cells/kg	IV	Recruiting
NCT06138210	Acute ischemic stroke	Within 7 days of symptom onset	Human induced pluripotent stem cells	3.0 mL, 1 × 10^11^ particles/mL	IV	Recruiting
NCT06612710	Ischemic stroke	Within 7 days of symptom onset	Human induced neural stem cells	4 × 10^9^ particles/kg	IV	Recruiting
NCT05158101	Stroke (unspecified)	NA	Allogeneic adult umbilical cord-derived mesenchymal stem cells	Approximately, 800 billion exosomes	IN	Recruiting
NCT06129175	Acute ischemic stroke	Within 4 weeks of symptom onset	Umbilical cord-derived mesenchymal stem cells	2 million UCMSCs per kg body weight	IV	Recruiting
NCT06299033	Chronic ischemic stroke	Between 6 months and 60 months after symptom onset	Human forebrain neural progenitor cells	1.5 × 10^7^ cells 3.0 × 10^7^ cells 6.0 × 10^7^ cells	IC	Recruiting
NCT04097652	Acute ischemic stroke	Within 48–168 h after symptom onset	Human umbilical cord derived-mesenchymal stem cells	NA	IV	Recruiting
NCT02795052	Ischemic stroke	At least 6 months after symptom onset	Autologous bone marrow derived stem cells	NA	IV and IN	Recruiting
NCT07084012	Acute ischemic stroke	Within 72 h of symptom onset	Human umbilical cord mesenchymal stem cells	1.0 × 10^8^ cells 2.0 × 10^8^ cells	IV	Recruiting
NCT07145294	Acute ischemic stroke	Within 2 weeks of symptom onset	Induced pluripotent stem cells-derived small extracellular vesicles	2 × 10^10^/mL, 2 mL	IN	Recruiting
NCT02605707	Chronic ischemic stroke	Within 6–60 months after symptom onset	Autologous endothelial progenitor cells	NA	IV	Completed
NCT01151124	Stable ischemic stroke	Within 6 months to 5 years after symptom onset	CTX0E03 DP, human neural stem cells product	2 million cells 5 million cells 10 million cells 20 million cells	IC	Completed
NCT03356821	Perinatal arterial ischemic stroke	Within 1 week of symptom onset	Bone marrow-derived allogeneic stem cells	50 × 10^6^ cells	IN	Completed
NCT00875654	Ischemic stroke	Within 6 weeks of symptom onset	Autologous mesenchymal stem cells	100 million cells; 300 million cells	IV	Completed
NCT01501773	Acute ischemic stroke	Within 7–30 days of symptom onset	Autologous bone marrow-derived stem cells	30–500 million cells	IV	Completed
NCT04811651	Ischemic stroke	Within 6 months of symptom onset	Umbilical cord-derived mesenchymal stem cells	One hundred million cells	IV	Completed
NCT00950521	Chronic ischemic stroke	Within 6–60 months of symptom onset	Autologous peripheral blood CD34 stem cells	2–8 million stem cells	IC	Completed
NCT01297413	Chronic ischemic stroke	Clinical diagnosis of ischemic stroke for longer than 6 months	Allogeneic adult mesenchymal bone marrow stem cells	0.5–1.5 million cells per kg	IV	Completed
NCT03080571	Subacute ischemic stroke	Within 14 days of the ictus	Autologous bone marrow derived mononuclear stem cells	1.59 × 10^8^ cells	IA	Completed
NCT01678534	Acute ischemic stroke	Within two weeks from the onset of stroke symptoms.	Allogenic mesenchymal stem cells from adipose tissue	1 million units/kg	IV	Completed
NCT02425670	Acute ischemic stroke	Onset from 7 to less than 30 days	Autologous bone marrow-derived stem cells	30–500 million cells	IV	Completed
NCT03004976	Acute ischemic stroke	Within 3–10 days following stroke.	Allogeneic umbilical cord blood hematopoietic stem cells	0.5 to 5 × 10^7^ total nucleated cell count (TNCC)/kg	IV	Completed
NCT02813512	Chronic ischemic stroke	Within 6 months to 10 years after disease onset	Human adipose-derived stem cells	1 × 10^8^ cells	IC	Completed
NCT05697718	Ischemic stroke	Within 12–24 weeks after the first symptom onset	Human umbilical cord mesenchymal stem cells	5.0 × 10^7^ cells 10.0 × 10^7^ cells 20.0 × 10^7^ cells	IV	Completed
NCT00473057	Acute and sub-acute ischemic stroke	>3 and <90 days after symptoms onset	Autologous bone marrow mononuclear cells	500 × 10^6^ cells	IA and IV	Completed
NCT00761982	Acute ischemic stroke	Within 5–9 days after disease onset	Autologous bone marrow-derived CD34+ stem cells	1.59 × 10^8^ BM-MNCs, of them, 3.38 × 10^6^ were CD34+ cells.	IA	Completed
NCT01287936	Chronic ischemic stroke	Within 6 months to 60 months after disease onset	SB623, modified bone marrow-derived mesenchymal stem cells	2.5 × 10^6^ cells 5 × 10^6^ cells 10 × 10^6^ cells	IC	Completed
NCT03371329	Acute spontaneous haemorrhagic stroke.	Within 72 h after stroke onset	Bone marrow derived cultured mesenchymal stem cell	0.5 × 10^6^ cells/kg 1 × 10^6^ cells/kg 2 × 10^6^ cells/kg 5 × 10^6^ cells/kg	IT and IV	Completed
NCT05292625	Ischemic stroke	Within 24 months after the ictus	Allogeneic umbilical cord mesenchymal stem cells	1.5 × 10^6^ cells/kg	IT and IV	Completed
NCT00535197	Acute ischemic stroke	Within 7 days of the ictus	Autologous bone marrow derived CD34+ stem cells	1.2 × 10^6^ cells 2.79 × 10^6^ cells 2.52 × 10^6^ cells 2.07 × 10^6^ cells 2.42 × 10^6^ cells	IA	Completed
NCT02117635	Chronic ischemic stroke	More than 3 months after the ictus	Allogeneic human neural stem cell	20 million cells	IC	Completed
NCT01468064	Acute ischemic stroke	Within 7 days of the onset of symptoms	Autologous bone marrow-derived mesenchymal stem cells	2.5 million cells per kg	IV	Completed
NCT02448641	Chronic ischemic stroke	Within the period of 6–90 months after the ictus	SB623, modified bone marrow-derived mesenchymal stem cells	2.5 million cells 5 million cells	IC	Completed
NCT02397018	Ischemic stroke	Within 3–10 days after the ictus	Allogeneic umbilical cord blood	0.5 to 5 × 10^7^ total nucleated cells /kg	IV	Completed
NCT04608838	Acute ischemic stroke	Within 48 h of after the ictus	JTR-161, allogeneic human dental pulp stem cell	1 or 3 × 10^8^ cells	IV	Completed
NCT02378974	Ischemic stroke	Within 7 days of the ictus	Human umbilical cord-derived mesenchymal stem cells	NA	IV	Completed
NCT06518902	Acute ischemic stroke	Within one month after the onset of stroke symptoms	Umbilical cord-derived mesenchymal stem cells	NA	NA	Not yet recruiting
NCT07197606	Subacute ischemic stroke	14–90 days after ischemic stroke.	Human umbilical cord-derived mesenchymal stem cells	NA	IV	Not yet recruiting
NCT06997939	Ischemic stroke	Between 3 and 12 months after the ictus	Autologous bone marrow mesenchymal stem cell	2.5 × 10^6^ cells 1.0 × 10^7^ cells 2.0 × 10^7^ cells	IA	Not yet recruiting
NCT07232563	Ischemic stroke	Within 24 h to 14 days of stroke onset	Human umbilical cord mesenchymal stem cell-derived small extracellular vesicles (hUC-MSC-sEV-001)	4 times, 1 × 10^11^ particles per dose	IN	Not yet recruiting
NCT07143786	Acute ischemic stroke	Within 1 week after symptom onset	Human induced neural stem cell-derived exosomes	5.0 × 10^11^ to 2.0 × 10^12^ particles	IV	Not yet recruiting
NCT06995625	Acute ischemic stroke	Within 5 days of symptom onset	Stem cell-derived extracellular vesicle	4.8 × 10^10^ particles 9.6 × 10^10^ particles 19.2 × 10^10^ particles	IV	Not yet recruiting
NCT03899298	Stroke (unspecific)	NA	Amniotic and umbilical cord stem cell	NA	NA	Not yet recruiting
NCT03725865	Haemorrhagic stroke	Between 12 and 60 months after the ictus	Human peripheral blood derived induced neural stem cells	NA	IC	Unknown status
*Chinese Clinical Trial Registry* (https://www.chictr.org.cn)						
ChiCTR2400090715	Acute ischemic stroke	Within 1 month after the ictus	Umbilical cord-derived mesenchymal stem cell	NA	NA	Recruiting
ChiCTR2400094054	Ischemic stroke	Between 3 and 60 months after stroke onset	Human neural stem cells	1 × 10^6^/200 μL/×2 5 × 10^6^/200 μL/×2 2.5 × 10^7^/500 μL/×2	IC and IN	Completed
ChiCTR2200056902	Ischemic stroke	Between 3 and 24 months after stroke onset	Human neural stem cells	NA	IC and IN	Not yet recruiting
ChiCTR1900027199	Haemorrhagic stroke	Between 6 months and 1 year after stroke onset	Neural stem cells	5 × 10^5^ cells 5 × 10^7^ cells	IN	Not yet recruiting
ChiCTR-ONRC-14005040	Acute ischemic stroke	Within 24–72 h after stroke onset	Autologous bone marrow mesenchymal stem cells	NA	NA	Recruiting
ChiCTR1900021987	Acute ischemic stroke	Within 72 h after stroke onset	Allogenic umbilical cord mesenchymal stromal cells	NA	NA	Not yet recruiting
ChiCTR-ONC-17014173	Ischemic stroke	NA	Neural stem cells	NA	NA	Recruiting
ChiCTR-INR-16008908	Ischemic stroke	Within 1–3 months after stroke onset	Allogenic bone marrow-derived mesenchymal stem cells	NA	IT	Recruiting
ChiCTR-ONRC-12002929	Ischemic stroke	More than 14 days after symptom onset	Umbilical cord mesenchymal stem cells	NA	IV and IT	Completed
ChiCTR2500095990	Subacute hemorrhagic stroke	Between 3 and 10 days after symptom onset	Human umbilical cord mesenchymal stem cells	NA	IV	Not yet recruiting
ChiCTR-TRC-14005071	Subacute ischemic stroke	Within 2 weeks after symptom onset	Umbilical cord blood stem cell	NA	NA	Recruiting
ChiCTR2200057960	Ischemic stroke	Between 72 h and 144 h after ictus	Human neural stem cells	NA	IA and IC and IN	Not yet recruiting
ChiCTR2300069718	Ischemic stroke	Within 12–24 weeks after symptom onset	Human umbilical cord mesenchymal stem cells	5.0 × 10^7^ cells 10.0 × 10^7^ cells 20.0 × 10^7^ cells	IV	Not yet recruiting
ChiCTR1900022741	Ischemic stroke	Between 3 months and 2 years after stroke onset	Human-derived neural stem cells	NA	IN	Recruiting
ChiCTR1800014354	Ischemic stroke	Between 4 months and 24 months after onset	Human neural stem cell	NA	IC	Recruiting
*EU Clinical Trials Register* (https://www.clinicaltrialsregister.eu)						
2012-003482-18	Ischemic stroke	NA	Human neural stem cell	20 × 10^6^ cells	IC	Completed
2011-003551-18	Acute ischemic stroke	Within two weeks after symptom onset	Allogeneic human adipose-derived stem cells	1 × 10^7^ cells	IV	Ongoing

**Table 3.** Preclinical studies.

Animals	Disease	Administration time	Classification of stem cells	Dose	Route	Reference
Rat	Hemorrhagic stroke	On the first day after stroke onset	Human neural stem cells	5 × 10^6^ cells	IV	Jeong et al. [9]
Rat	Ischemic stroke	On the 7th day after stroke onset	Bone marrow stromal cells	1 × 10^6^ cells, IC 3 × 10^6^ cells, IV	IC and IV	Kawabori et al. [57]
Mouse	Hemorrhagic stroke	At 1 week after stroke onset	Human neural stem cells	2 × 10^5^ cells	IC	Lee et al. [58]
Rat	Hemorrhagic stroke	At 2 or 24 h after stroke onset	Human neural stem cells	5 × 10^6^ cells, IV 1 × 10^6^ cells, IC	IV and IC	Lee et al. [10]
Rat	Hemorrhagic stroke	At 2 h after stroke onset	Mesenchymal stem cells	5 × 10^6^ cells	IV	Chen et al. [12]
Monkey	Hemorrhagic stroke	At either 1 or 4 weeks after stroke onset	Human mesenchymal stem cells	(1–5) × 10^6^ cells	IC	Feng et al. [62]
Rat	Hemorrhagic stroke	At 30 min after stroke onset	Bone marrow-mesenchymal stem cells	1 × 10^5^ cells	IT	Wang et al. [37]
Rat	Hemorrhagic stroke	At 2 days after stroke onset	Human umbilical cord blood-derived mesenchymal stem cells	1 × 10^5^ cells	IT	Ahn et al. [38]
Rat	Hemorrhagic stroke	At 1 h after stroke onset	Human umbilical cord mesenchymal stromal cells	3 × 10^6^ cells	IV	Mello et al. [13]
Rat	Hemorrhagic stroke	At 2 days after stroke onset	Human umbilical cord blood-derived mesenchymal stem cells	1 × 10^5^ cells	IT	Ahn et al. [49]
Rat	Hemorrhagic stroke	At 48 h after stroke onset	Adipose-derived stem cells	2–4 × 10^7^ cells	IT	Chen et al. [50]
Rat	Hemorrhagic stroke	At 24 h after stroke onset	Human adipose-derived stem cells	1 × 10^6^ cells	IV	Yang et al. [14]
Rat	Hemorrhagic stroke	At 48 h after stroke onset	Adipose-derived mesenchymal stromal cells	3–4 × 10^5^ cells	IC	Zhang et al. [63]
Rabbit	Hemorrhagic stroke	At 24 h after stroke onset	Human amniotic epithelial cells	2 × 10^6^ cells	IT	Zhou et al. [51]
Rat	Hemorrhagic stroke	At 24 h after stroke onset	Human amniotic membrane mesenchymal stem cells	5 × 10^5^ cells	IC	Zhou et al. [64]
Mice	Hemorrhagic stroke	At 24 h after stroke onset	Human amnion-derived stem cells; Human adipose-derived stem cells	1.0 × 10^5^ cells 2.5 × 10^4^ cells 1.0 × 10^6^ cells	IV	Kuramoto et al. [15]
Rat	Hemorrhagic stroke	At 24 h after stroke onset	Induced pluripotent stem cells	1 × 10^6^ cells	IC	Qin et al. [65]
Rat	Hemorrhagic stroke	At 6 h after stroke onset	Induced pluripotent stem cells	1 × 10^6^ cells	IC	Qin et al. [66]
Rat	Ischemic stroke	Three days following stroke onset	Human bone marrow-derived mesenchymal stem cells	2.5 × 10^5^ cells	IV and IA	Byun et al. [24]
Mice	Hemorrhagic stroke	At 3 and 7 days after stroke onset	Hypoxic preconditioned bone marrow-derived mesenchymal stem cells	1 × 10^6^ cells	IN	Sun et al. [66,71]
Mice	Ischemic stroke	On day 1, day 3 and day 7 after stroke onset	Human neural stem cell	1 × 10^6^ cells 1 × 10^5^ cells	IV, IN and IT	Zhang et al. [70]
Mice	Multiple sclerosis	At the end of the fourth week of cuprizone administration.	Bone marrow mesenchymal stem cells	2 × 10^6^ cells	IP	Marzban et al. [111]

## Data Availability

Data sharing is not applicable to this article as no data were created or analysed in this study.
